# The Novel Artemisinin Dimer Isoniazide ELI-XXIII-98-2 Induces c-MYC Inhibition, DNA Damage, and Autophagy in Leukemia Cells

**DOI:** 10.3390/pharmaceutics15041107

**Published:** 2023-03-30

**Authors:** Mohamed Elbadawi, Joelle C. Boulos, Mona Dawood, Min Zhou, Waseem Gul, Mahmoud A. ElSohly, Sabine M. Klauck, Thomas Efferth

**Affiliations:** 1 Department of Pharmaceutical Biology, Institute of Pharmaceutical and Biomedical Sciences, Johannes Gutenberg University-Mainz, 55128 Mainz, Germany; 2 Department of Molecular Biology, Faculty of Medical Laboratory Sciences, Al-Neelain University, Khartoum 12702, Sudan; 3 ElSohly Laboratories, Inc., 5 Industrial Park Drive, Oxford, MS 38655, USA; 4 Division of Cancer Genome Research, German Cancer Research Center (DKFZ), German Cancer Consortium (DKTK), National Center for Tumor Diseases (NCT), 69120 Heidelberg, Germany

**Keywords:** artemisinin, cell death, chemotherapy, leukemia, oncogenes, sesquiterpenoids

## Abstract

The proto-oncogenic transcription factor c-MYC plays a pivotal role in the development of tumorigenesis, cellular proliferation, and the control of cell death. Its expression is frequently altered in many cancer types, including hematological malignancies such as leukemia. The dimer isoniazide ELI-XXIII-98-2 is a derivative of the natural product artemisinin, with two artemisinin molecules and an isoniazide moiety as a linker in between them. In this study, we aimed to study the anticancer activity and the molecular mechanisms of this dimer molecule in drug-sensitive CCRF-CEM leukemia cells and their corresponding multidrug-resistant CEM/ADR5000 sub-line. The growth inhibitory activity was studied using the resazurin assay. To reveal the molecular mechanisms underlying the growth inhibitory activity, we performed in silico molecular docking, followed by several in vitro approaches such as the MYC reporter assay, microscale thermophoresis, microarray analyses, immunoblotting, qPCR, and comet assay. The artemisinin dimer isoniazide showed a potent growth inhibitory activity in CCRF-CEM but a 12-fold cross-resistance in multidrug-resistant CEM/ADR5000 cells. The molecular docking of artemisinin dimer isoniazide with c-MYC revealed a good binding (lowest binding energy of −9.84 ± 0.3 kcal/mol) and a predicted inhibition constant (pKi) of 66.46 ± 29.5 nM, which was confirmed by microscale thermophoresis and MYC reporter cell assays. Furthermore, c-MYC expression was downregulated by this compound in microarray hybridization and Western blotting analyses. Finally, the artemisinin dimer isoniazide modulated the expression of autophagy markers (LC3B and p62) and the DNA damage marker pH2AX, indicating the stimulation of both autophagy and DNA damage, respectively. Additionally, DNA double-strand breaks were observed in the alkaline comet assay. DNA damage, apoptosis, and autophagy induction could be attributed to the inhibition of c-MYC by ELI-XXIII-98-2.

## 1. Introduction

Leukemia represents a group of life-threatening hematological malignancies that are characterized by elevated leukocyte counts in the blood and bone marrow. Leukemia comprises several diverse subgroups which vary according to their origin, pathogenesis, incidence, and prognosis [1]. The predominant leukemia cell populations might be more differentiated cells, as in chronic lymphocytic leukemia (CLL), precursor cells of diverse origin, as in acute leukemias, or both precursor and mature cells, as in chronic myeloid leukemia (CML) [2,3]. As of the year 2020, leukemia was responsible for 474,519 new cases and 311,594 deaths worldwide [4]. According to the statistics of the National Cancer Institute (USA), 60,650 new cases were reported in 2022, and the number of estimated deaths was 24,000 in the US [5].

Natural products play a pivotal role in the treatment of various diseases. In the field of cancer drug discovery, more than 60% of the anticancer agents are directly or indirectly derived from a natural origin [6]. Artemisinin (ART) is a sesquiterpene lactone extracted from sweet wormwood (*Artemisia annua* L.), which has been used in ancient Chinese medicine for thousands of years. As a potent antimalarial drug, artemisinin belongs to the standard protocols for the treatment of malaria caused by *Plasmodium* parasites [7,8]. 

Over the past three decades, artemisinin and its derivatives have been recognized as potential antitumor agents in vivo and in vitro [9,10]. Artemisinin was proven to have multiple mechanistic anticancer activities on different types of tumors [11]. Interestingly, artemisinin and its semisynthetic derivatives induce cell death, DNA damage, oxidative stress, and cell cycle arrest in cancer cells [12,13,14,15]. Moreover, they inhibit different cancer-associated signaling pathways, angiogenesis, invasion, and metastasis [16,17,18,19,20]. Recently, dimer derivatives of artemisinin have been developed, and they showed promising anticancer and antimalarial activities. Some of these dimers are more potent than artemisinin itself [21,22].

c-MYC is a nuclear transcription factor of the basic-helix-loop-helix-leucine zipper family. It is a 62 kDa protein with 439 residues and several functional domains which are essential for its interactions with DNA, other transcription factors, and cellular components. It plays an essential role in regulating the cell cycle, differentiation, metabolism, angiogenesis, immune response, DNA repair, and apoptosis [23,24,25]. Due to these essential roles, the deregulation of c-MYC expression is thought to be involved in many types of cancers. The deregulation of c-MYC expression can be caused by gene amplification, somatic mutations, transcriptional and post-transcriptional regulation, and translational and post-translational modifications [26].

Although initially regarded as undruggable, MYC inhibitor development has recently progressed significantly. In fact, several compounds that either directly or indirectly target c-MYC exert anticancer properties in preclinical tumor models [27].

This study aims to investigate the anticancer activity of the novel artemisinin dimer isoniazide ELI-XXIII-98-2 using sensitive and multidrug-resistant leukemia cell lines and to explore the molecular mechanism of its anticancer activity.

## 2. Materials and Methods

### 2.1. Compounds

**Figure 1:**
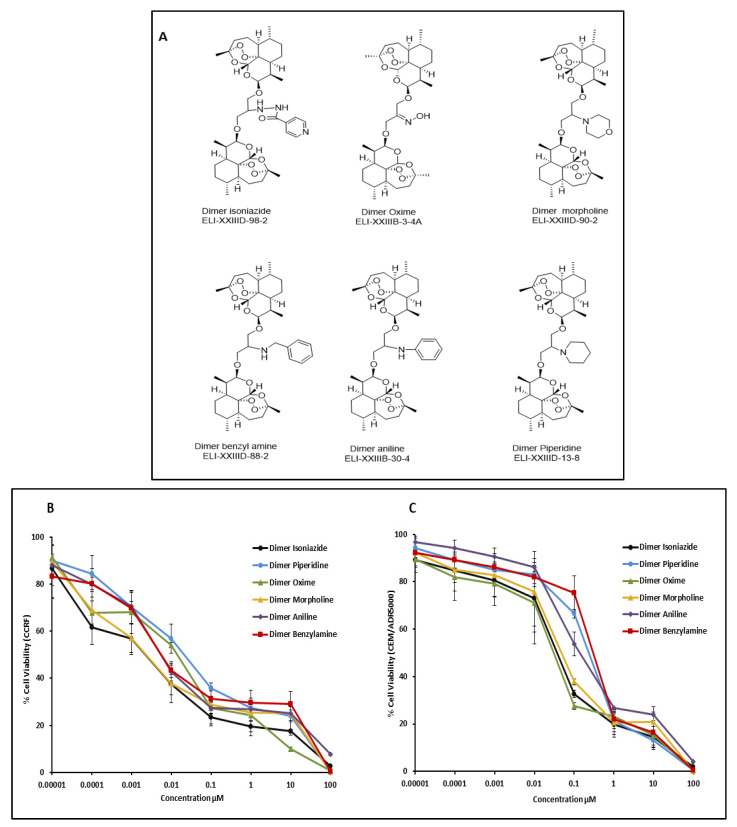
(**A**) Chemical structures of artemisinin dimers. (**B**) Growth inhibitory activity of six artemisinin dimers towards CCRF-CEM leukemia cells. (**C**) Growth inhibitory activity of six artemisinin dimers towards multidrug-resistant CEM/ADR5000 cells. Data are expressed as mean values ± SD of three independent experiments.

The artemisinin dimers were synthesized and provided by El Sohly Laboratories, Inc. (Oxford, MS, USA) (Figure 1A and Table 1). The stock solutions were prepared in DMSO at a final concentration of 20 mM. 

### 2.2. Cell Lines

We evaluated the activity of artemisinin dimers against the drug-sensitive leukemic CCRF-CEM cell line and its corresponding multidrug-resistant CEM/ADR5000 subline. The cell lines were obtained from Dr. Axel Sauerbrey (University of Jena, Jena, Germany). The multidrug resistance phenotype was previously characterized [28,29,30]. Both cell lines were cultured in RPMI medium supplemented with 10% FBS and 1% penicillin/streptomycin (Invitrogen, Darmstadt, Germany). The cells were incubated at 37 °C in a humidified atmosphere and 5% CO_2_.

### 2.3. Cell Viability Assay

The viability-inhibiting activity of artemisinin dimers was assessed using a resazurin reduction assay [31]. Both cell lines were treated with increasing concentrations of the dimers (from 10^−5^ to 100 µM) and incubated for 72 h before resazurin (Promega, Mannheim, Germany) was added. Briefly, resazurin is reduced by living cells to its fluorescent metabolite resorufin, while the dead cells are not able to metabolize resazurin. The fluorescence was detected by an Infinite M2000 reader (Tecan, Grailsheim, Germany). Each experiment was conducted thrice, each with six replicates. The dose–response curves were generated, and the 50% inhibitory concentrations (IC_50_) were calculated using Microsoft Excel 2019.

### 2.4. Molecular Docking

Molecular docking was used to study the in silico binding of the artemisinin dimer isoniazide to c-MYC. The crystalline structure of the c-MYC was retrieved from the Protein Data Bank (https://www.rcsb.org/) (accessed on 27 September 2022) and then refined using AutoDock Tools-1.5.6rc3 (https://autodock.scripps.edu/, California, USA) [32,33]. In each structure, water molecules and other ligands were removed, missing hydrogen atoms were added, and the structure was finally saved as a PDBQT format. The structures of ligands were prepared and saved as a PDBQT format as well. Molecular docking was performed using AutoDock 4.2.6, and the Lamarckian algorithm was applied. The docking parameters were adjusted to 250 runs and 2,500,000 energy evaluations. The results obtained from the DLG files were expressed as binding energies and molecular interactions [34].

### 2.5. Microscale Thermophoresis

The in vitro binding of the artemisinin dimer isoniazide to human c-MYC was studied by microscale thermophoresis (MST). Recombinant human c-MYC protein (ab169901) was purchased from Abcam (Cambridge, UK) at a concentration of 0.5 mg/mL. In brief, the c-MYC protein was fluorescently tagged using the Monolith NTTM Protein Labeling Kit Blue (L003, NanoTemper Technologies GmbH, Munich, Germany). For this experiment, Monolith NT.115 system and Monolith NT.115 standard capillaries (MO-K022) were used. Afterward, MST was conducted at 20% LED power and 40% MST power at a final protein concentration of 200 nM. Finally, NanoTemper Analysis Software (version 1.5.41) was used to fit the data according to the law of mass action, and the dissociation constant (K_d_) for the artemisinin dimer isoniazide was calculated using the following equation:

$$f\left(c\right)=\frac{c+c_{T}+K_{d}-\sqrt{{\left(c+c_{T}+K_{d}\right)}^{2}+4\;c\;c_{T}}}{2\;c_{T}}$$

where f(c) is the fraction bound, c is the ligand concentration, and C_T_ is the protein concentration.

### 2.6. MYC Reporter Cell Assay

To study the effect of the artemisinin dimer isoniazide on MYC activity, a signal MYC reporter assay (CCS-012L, Qiagen, Germantown, MD, USA) was performed. Briefly, human embryonic kidney HEK293 cells were transiently transfected with a c-MYC-luciferase reporter construct provided in the kit. The cells were then cultured following the manufacturer’s guidance. Afterward, cells were subjected to different concentrations of artemisinin dimer isoniazide (2, 5, and 10 µM) 10058-F4 (127.5 µM) as a positive control or DMSO as a negative control for 48 h. A Dual-glo^®^ Luciferase Reporter Assay System (E2920, Promega, Madison, WI, USA) was used to measure the c-MYC promoter activity by quantifying renilla and firefly luciferase luminescence with an Infinite M2000 Pro™ plate reader (Tecan). The c-MYC activity was obtained by calculating the ratio of the firefly luciferase luminescence to the renilla luciferase luminescence. The relative luminescence was obtained by multiplying the c-MYC activity by 1000. Finally, the normalized c-MYC activity was obtained by calculating the ratio of the relative luciferase luminescence of the sample to the relative luciferase luminescence of the DMSO.

### 2.7. Microarray Analyses

We performed microarray hybridization expression analyses to reveal the molecular mechanisms related to the anticancer activity of the artemisinin dimer isoniazide. CCRF-CEM cells were treated with 2.5 µM of the dimer for 24 h, and the total RNA was subsequently extracted using an InviTrap^®^ Spin Universal RNA Mini Kit (Invitek Molecular, Berlin, Germany). Afterward, complementary DNA (cDNA) was synthesized and labeled, and hybridization was then performed on Affymetrix GeneChips^®^ with the human Clariom S™ assay (Affymetrix, Santa Clara, CA, USA) at the Genomics and Proteomics Core Facility of the German Cancer Research Center (DKFZ, Heidelberg, Germany). Data analysis was then conducted using Chipster software (http://chipster.csc.fi/) (version 3.16.3) (accessed on 21 June 2020) to sort out the affected genes according to their variable expression and significance based on the empirical Bayes t-test (*p* < 0.05). To reveal the cellular functions and pathways affected by the artemisinin dimer isoniazide, the genes were further analyzed using Ingenuity Pathway Analysis software (content version: 51963813, Release Date: 11/3/2020) (IPA; Ingenuity Systems, Redwood City, CA, USA).

### 2.8. Quantitative Real-Time PCR (qPCR)

To validate the microarray findings, we performed a qPCR with selected genes. In brief, the CCRF-CEM cells were treated with the artemisinin dimer isoniazide (2.5 and 5 µM) or DMSO as a negative control for 24 h. Afterward, the total RNA was extracted using an InviTrap^®^ Spin Universal RNA Mini Kit (Invitek Molecular, Berlin, Germany), as mentioned above. Next, 1 µg RNA was converted into cDNA using the LunaScript^®^ RT SuperMix Kit cDNA Synthesis Kit (New England Bio Labs, Darmstadt, Germany). Subsequently, the genes were amplified using an Eva green master mix (5× Hot Start Taq EvaGreen^®^ qPCR Mix (no ROX); Axon Labortechnik, Kaiserslautern, Germany), according to the manufacturer’s instructions. PCR primers were designed by the Primer-BLAST online tool (https://www.ncbi.nlm.nih.gov/tools/primer-blast/) (accessed on 2 January 2023) and purchased from Eurofins Genomics (Ebersberg, Germany). Sequences of the selected genes and GAPDH (control) are shown in Table 2. Using 38 well plates, 40 cycles of real-time PCR were conducted on CFX384TM (Bio-Rad, Munich, Germany). The run conditions included a 15 s denaturation phase at 95 °C, followed by a 30 s gradient annealing step at 62–47 °C and finally a 1 min elongation step at 72 °C. Bio-CFX Rad’s Manager Software (version 3.1) was used to determine the Cq values. The comparative Cq (2^−ΔΔCq^) method was used to determine the gene expression fold-change [35].

### 2.9. Detection of Apoptosis by Flow Cytometry

An annexin V-FITC/PI double-staining kit (Invitrogen, Life Technologies GmbH, Darmstadt, Germany) was used for apoptosis detection. In brief, CCRF-CEM cells were seeded in 6 well plates at a density of 1 × 10^6^ cells/well. They were then treated with increasing concentrations of the artemisinin dimer isoniazide (2.5, 5, and 10 µM) or DMSO as a negative control and incubated for 48 h. Afterward, the cells were harvested, washed, and resuspended in 1 mL cold PBS combined with 500 µL binding buffer. Subsequently, the cells were stained using 5 µL annexin V-FITC for 15 min, followed by the addition of 10 µL of propidium iodide (PI) for 15 min at room temperature in the dark. Finally, apoptosis was detected using a flow cytometer (BD Accuri™ C6, BD Biosciences, Becton Drive, Franklin Lakes, NJ, USA). The signal detector for FITC is FL1, while for PI it is FL2. This experiment was repeated three times.

### 2.10. Immunoblotting

The CCRF-CEM cells were treated with increasing concentrations of the dimer isoniazide for 24 h. Afterward, the total protein was extracted using an M-PER^®^ protein extraction reagent (Thermo Scientific, Darmstadt, Germany) supplemented with a cocktail of protease and phosphatase inhibitors. In brief, 30 mg of the extracted proteins were denatured after the addition of β-mercaptoethanol and then heated at 95 °C for 10 min. Afterward, protein separation was carried out by sodium dodecyl sulfate-polyacrylamide gel electrophoresis (SDS-PAGE). The separated proteins were transferred onto polyvinylidene difluoride (PVDF) membranes. The membranes were washed with Tris-buffered saline containing 0.5% Tween-20 (TBST) and incubated with a blocking buffer (5% (*w*/*v*) bovine serum albumin in TBST) for 1 h at room temperature. Following a wash step, the membranes were incubated with primary antibodies at 4 °C overnight. The following antibodies were used: c-MYC, LC3B, p62, phospho-H2AX, and β-actin in a dilution of 1:1000 (Cell Signaling Technology, Frankfurt a. M., Germany). After washing the membranes three times with TBST, a horseradish-peroxidase-linked IgG secondary antibody was added and incubated for 2 h, followed by a washing step. Lastly, a Luminata™ Classico Western HRP substrate (Merck Millipore, Schwalbach, Germany) was added to the membranes for 3 min in the dark. The protein bands were visualized using an Alpha Innotech FluorChem Q system (Biozym, Oldendorf, Germany).

### 2.11. Alkaline Comet Assay

This assay was performed to detect DNA damage in the CCRF-CEM cells after treatment with the artemisinin dimer isoniazide. For this assay, an Oxiselect™ Comet Assay Kit (Cell Biolabs, San Diego, CA, USA) was used. In brief, the CCRF cells were seeded in 6 well plates at a density of 1 × 10^6^ per well. They were then treated with increasing concentrations of the artemisinin dimer isoniazide (1, 2.5, and 5 µM) with DMSO as negative control and H_2_O_2_ (50 µM) as a positive control (for 1 h). Afterward, the cells were harvested, centrifuged at 3000× *g* for 10 min, and resuspended in PBS. Subsequently, cell suspensions at a density of 1 × 10^5^ cells/mL were mixed with melting agarose at 37 °C at a ratio of 1:6. The mixtures were spread on comet slides and incubated in the dark at 4 °C for 30 min. The slides were then submerged in a pre-chilled lysis buffer (NaCl 14.6 g, EDTA solution 20 mL, 10× lysis solution, pH 10.0) for 1 h at 4 °C in the dark. Consequently, the slides were removed from the lysis buffer and immersed in a pre-chilled alkaline electrophoresis solution buffer (NaOH 12 g, EDTA solution 2 mL, 1000 mL distilled water) for 40 min at 4 °C in the dark. The slides were then placed in an electrophoresis chamber filled with an alkaline electrophoresis solution buffer, and the electrophoresis was run at 20 V for 20 min. The slides were then washed twice with prechilled distilled water for 5 min each. Next, the slides were immersed in cold ethanol (70%) for 5 min and allowed to air-dry. After complete dryness, 100 µL Vista Green DNA dye diluted 1:10,000 in TE buffer (10 mM Tris, 1 mM EDTA, pH 7.5) was added to each slide and incubated for 15 min at room temperature. Finally, images were obtained using an EVOS digital inverted microscope (Life Technologies GmbH, Darmstadt, Germany) and prepared using Image J software (version 1.53t). 

## 3. Results

### 3.1. Growth Inhibition Assay

The artemisinin dimers demonstrated potential inhibitory activity against both leukemic cell lines. The dimer isoniazide demonstrated the lowest IC_50_ value for CCRF-CEM, which was 2.5 ± 1.5 nM. For the CEM/ADR5000 subline, the lowest calculated IC_50_ was 29 ± 14 nM, which is attributed to the dimer oxime. The observed degree of resistance towards artemisinin dimers in the two cell lines ranged from 2.1–49.2 for the dimers oxime and dimer benzylamine, respectively. The dose–response curves are shown in Figure 1B,C, while the IC_50_ values and degree of resistance are shown in Table 1.

Since all dimers were cross-resistant in the CEM/ADR5000 cells, further investigations were conducted on CCRF-CEM using the artemisinin dimer isoniazide as it showed the best inhibitory activity with the lowest IC_50_ value for the CCRF-CEM cells.

### 3.2. Molecular Docking

**Figure 2:**
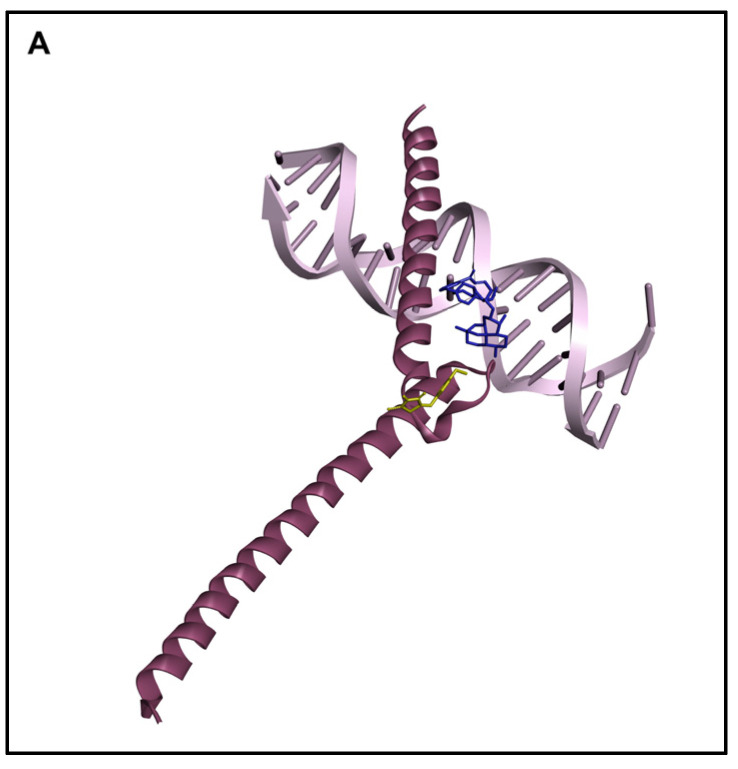

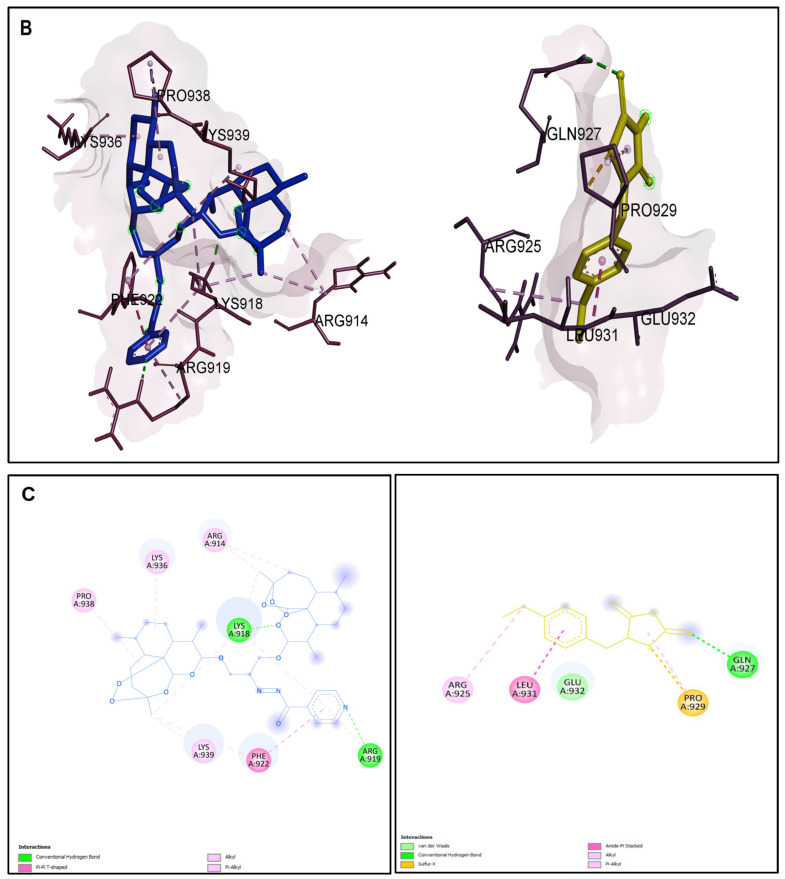
(**A**) Molecular docking of the dimer isoniazide and the known inhibitor 10058-F4 with c-MYC. The binding of 10058-F4 was confined more to the c-MYC and MAX interaction site, while the dimer isoniazide was closer to the DNA binding site. (**B**) Three-dimensional representation of the docking interactions of the dimer isoniazide (blue) and the known inhibitor 10058-F4 (yellow). (**C**) Two-dimensional representation of the docking interactions of the dimer isoniazide (blue) and the known inhibitor 10058-F4 (yellow).

A molecular docking study was performed to show the binding affinity of the artemisinin dimer isoniazide to c-MYC in silico. This compound showed a strong interaction, demonstrating the lowest binding energy of −9.84 ± 0.3 kcal/mol and a predicted inhibition constant (pKi) of 66.46 ± 29.5 nM. Interestingly, this binding energy was better than that of the known c-MYC inhibitor 10058-F4, which demonstrated a binding energy of −4.92 ± 0.01 kcal/mol and a pKi of 248.39 ± 3.51 nM. The docking results are visualized in Figure 2 and listed in Table 3.

### 3.3. Microscale Thermophoresis

**Figure 3:**
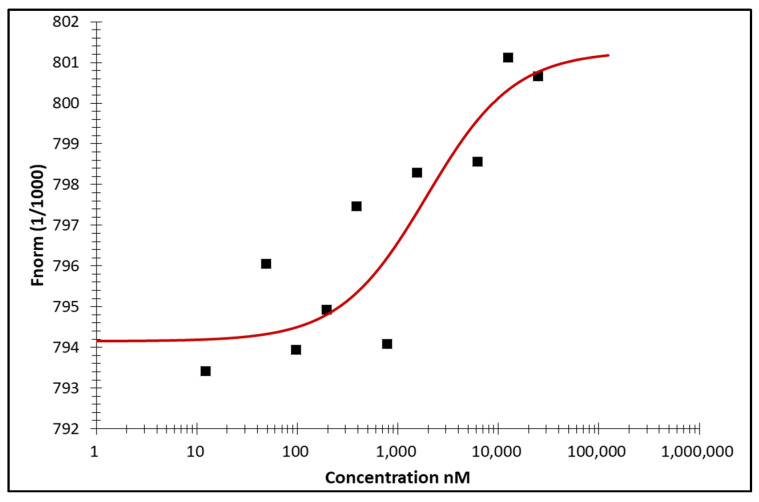
Binding of artemisinin isoniazide dimer to human c-MYC, as detected by microscale thermophoresis. The binding curve shows a concentration-dependent change in the fluorescence signal. Data fitting was achieved according to the law of mass action. K_d_ = 1.19 ± 0.24 µM, calculated after three independent repetitions.

Microscale thermophoresis (MST) was performed to study the in vitro binding of the artemisinin dimer isoniazide to the recombinant human c-MYC protein. MST revealed a concentration-dependent change in the fluorescence signal, showing a binding interaction between the compound and the protein. According to the law of mass action, the calculated dissociation constant (K_d_) was 1.19 ± 0.24 µM. The MST binding curve is shown in Figure 3.

### 3.4. MYC Reporter Cell Assay

**Figure 4:**
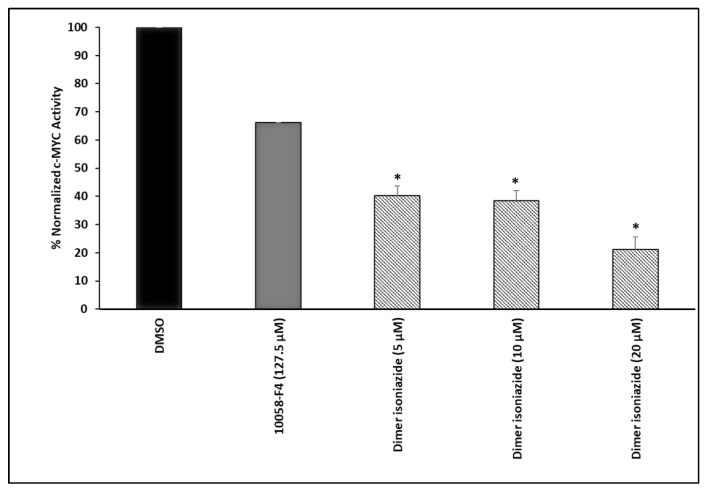
c-MYC reporter assay. Artemisinin isoniazide dimer significantly reduced the activity of c-MYC in a dose-dependent manner. The inhibitory effect was stronger than that of the known inhibitor 10058-F4 (127.5 µM). Both compounds were incubated for 48 h. Statistical significance in comparison to the control (DMSO) was designated at a level of * *p* ≤ 0.05.

This assay was conducted to study the effect of the artemisinin dimer isoniazide on the transcriptional activity of c-MYC. Treatment with an increasing concentration of the compound significantly decreased the activity of c-MYC in a dose-dependent manner. However, the known inhibitor 10058-F4 showed a weaker activity, even at a high concentration of 127.5 µM. The results are shown in Figure 4. These findings were in accordance with the data obtained from molecular docking and MST and as shown below by Western blotting.

### 3.5. Microarray Analyses

**Figure 5:**
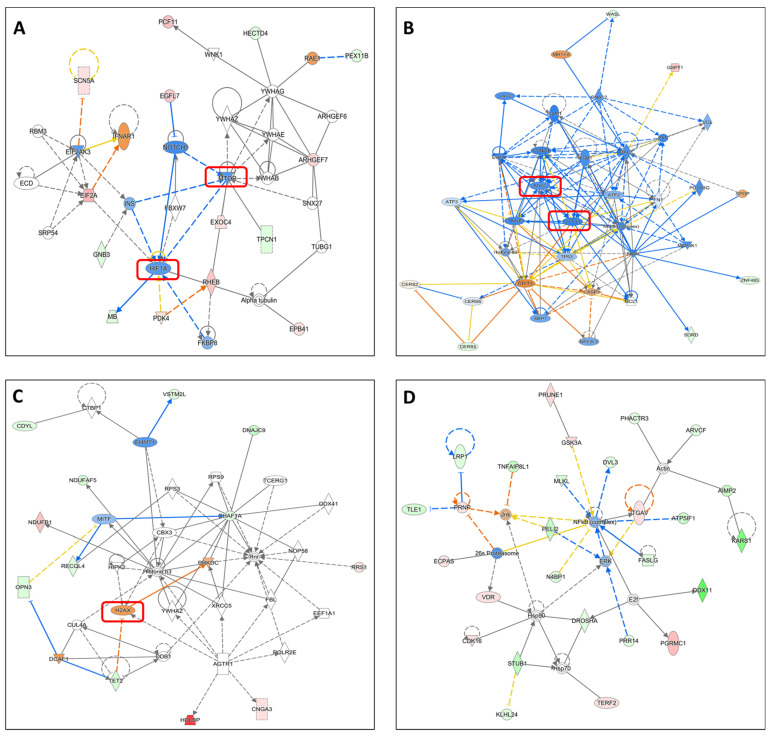
Microarray analyses. (**A**–**D**): Altered cellular networks in CCRF-CEM cells incubated with 2.5 μM dimer isoniazide. The compound downregulates the expression of several cancer-associated factors, such as *MYC*, *HIF1A*, *STAT3*, and *MTOR*, while increasing the expression of others, such as *H2AX*. Blue and green ions indicate inhibition or downregulation 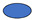 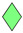. Red icons indicate activation or upregulation 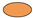 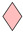.

Microarray expression analyses were conducted to uncover the molecular mechanisms underlying the growth inhibitory activity of the artemisinin dimer isoniazide. Here, we did not observe any potentially affected canonical pathway. Therefore, we investigated the cellular networks that could be modulated by the artemisinin dimer isoniazide. Treatment with the artemisinin dimer isoniazide downregulated several signal transducers and transcription factors such as *HIF1A*, *STAT3*, *MTOR*, and *NOTCH1* (Figure 5A,B), which are known to be involved in the process of tumorigenesis. Interestingly, the MYC gene was also downregulated (Figure 5B), a finding that fits the binding of the dimer isoniazide to the c-MYC protein in the molecular docking analysis, MST, and MYC reporter cell assay. Moreover, *H2AX*, the gene that encodes H2A histone family member X, and its interacting *PRKDC* gene encoding the catalytic subunit of DNA-activated protein kinase, which are both markers for DNA double-strand breaks, were upregulated by the artemisinin dimer isoniazide (Figure 5C). Additionally, microarray analyses revealed an upregulation of the *DDIT3* gene encoding DNA-damage-inducible transcript 3, which leads to apoptosis induction upon activation. *DDIT3* interacts with the apoptosis marker *CASP3*, which was also upregulated (Figure 5B). This points toward an activation of DNA double-strand breaks and damage. Furthermore, the dimer isoniazide downregulates the expression of *ERK* as well as the NF-κB complex, which are both essential for tumor progression (Figure 5D). 

### 3.6. Quantitative Real-Time PCR (qPCR)

**Figure 6:**
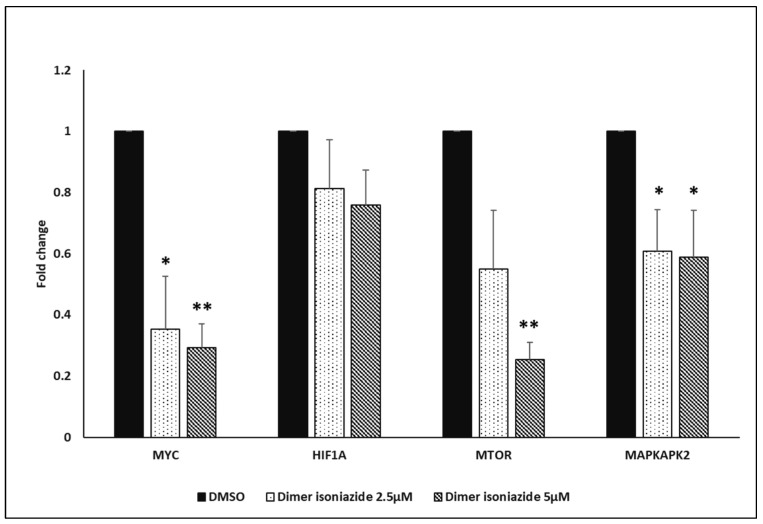
qPCR analysis. Treatment of CCRF-CEM cells with 2.5 and 5 µM of artemisinin isoniazide dimer significantly decreased the expression of *MYC*, *MTOR*, and *MAPKAPK2* genes, while no significant inhibition was observed for *HIF1A*. Statistical significance in comparison to control (DMSO) is designated at a level of * *p* ≤ 0.05 and ** *p* ≤ 0.01.

A qPCR was performed to confirm the finding of the microarray analyses. Treatment with the artemisinin dimer isoniazide (2.5 and 5 µM) significantly downregulated the mRNA expression of *MYC* in a dose-dependent manner. The *p*-values were ≤ 0.05 for the first concentration (2.5 µM), while the *p*-value was ≤ 0.01 for 5 µM. This finding was in parallel with those of the microarray analyses, MST, and MYC reporter cell assay. Similar effects were observed for the *MAPKAPK2* gene, with *p*-values ≤ 0.05 for both concentrations. The *MTOR* gene was significantly downregulated at a concentration of 5 µM (*p* ≤ 0.01). No significant inhibition was observed for *HIF1A*. The qPCR results are shown in Figure 6.

### 3.7. Detection of Apoptosis by Flow Cytometry

**Figure 7:**
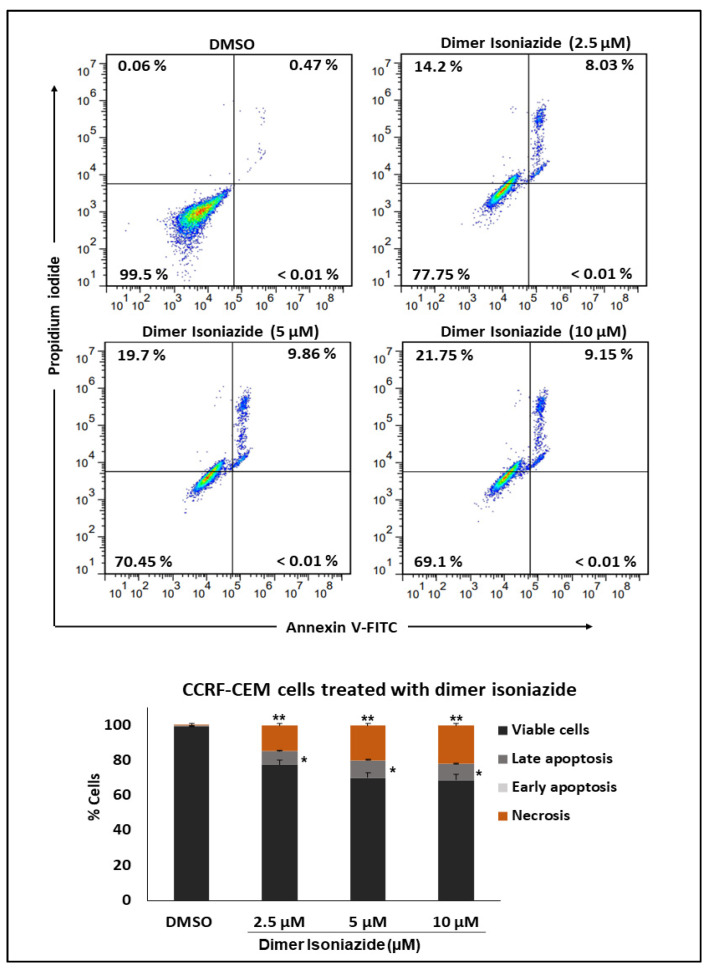
Detection of apoptosis by flow cytometry. Treatment with increasing concentrations of artemisinin dimer isoniazide significantly increased the percentage of necrotic as well as late apoptotic cells, while no early apoptosis was detected. Statistical significance in comparison to control (DMSO) is designated at a level of * *p* ≤ 0.05 and ** *p* ≤ 0.01.

Treatment with increasing concentrations of the artemisinin dimer isoniazide (2.5, 5, and 10 µM) showed a significant and dose-dependent increase of the necrotic cell fractions (*p* ≤ 0.01). On the other hand, we observed late apoptotic cells whose levels were less than those of the necrotic cells. Nevertheless, the late apoptotic cell fractions were still significant in comparison to the control (*p* ≤ 0.05). Early apoptotic cells were not detected. The results are shown in Figure 7.

### 3.8. Immunoblotting

Immunoblotting was performed to study the expression of c-MYC, autophagy markers (p62 and LC3B), and p-H2AX. Treatment with the artemisinin dimer isoniazide significantly reduced the expression of c-MYC (*p* ≤ 0.01 for the first three concentrations and *p* ≤ 0.001 for a concentration of 20 µM). Additionally, we detected a significant upregulation of p-H2AX in a dose-dependent manner. The *p*-values were ≤ 0.05 for the first concentration (2.5 µM), while the *p*- value was ≤ 0.01 for the higher concentration.

**Figure 8:**
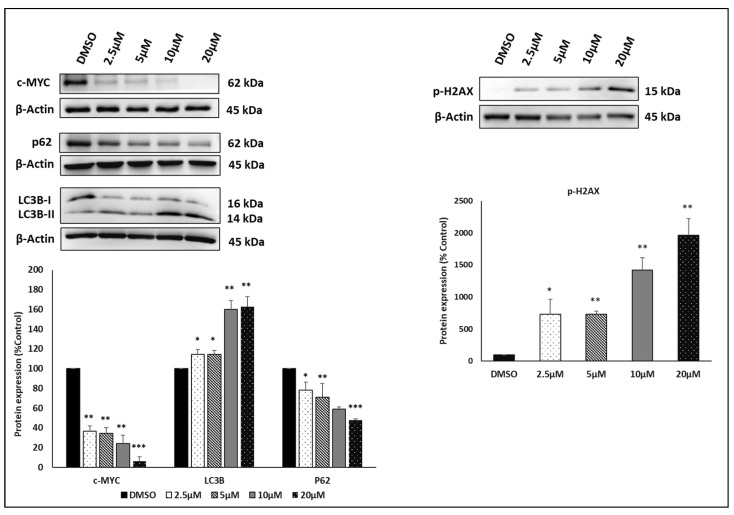
Immunoblot analysis. Treatment of CCRF-CEM cells with artemisinin dimer isoniazide significantly decreased the expression of c-MYC and p62 in a dose-dependent manner while increasing the expression of p-H2AX and the autophagy marker LC3B-II. Statistical significance in comparison to control (DMSO) is designated at a level of * *p* ≤ 0.05, ** *p* ≤ 0.01, and *** *p* ≤ 0.001.

To study the induction of autophagy, we examined the expression of two autophagy markers, p62 and LC3B. Treatment with the artemisinin dimer isoniazide significantly increased the expression of LC3B-II. The *p*-values were ≤ 0.05 for the lower concentrations (2.5 and 5 µM) and ≤ 0.01 for the higher concentrations (10 and 20 µM). On the other hand, the artemisinin dimer isoniazide significantly downregulated the expression of p62 in a dose-dependent manner (*p* ≤ 0.05 for 2.5 µM and *p* ≤ 0.01 and *p* ≤ 0.001 for 10 µM and 20 µM, respectively). The immunoblotting results are shown in Figure 8.

### 3.9. Alkaline Comet Assay

**Figure 9:**
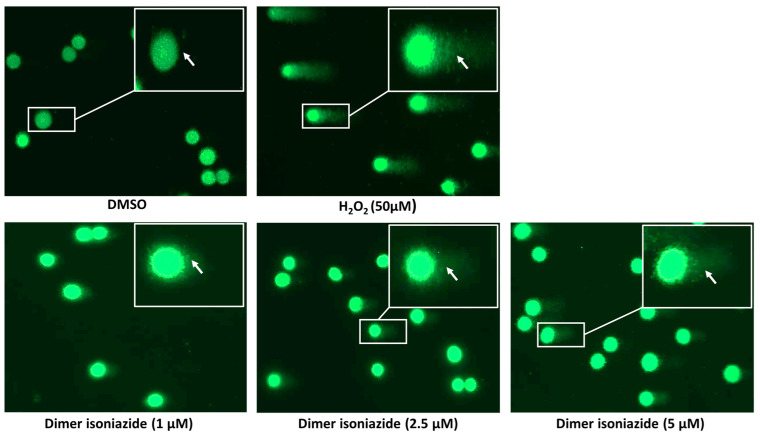

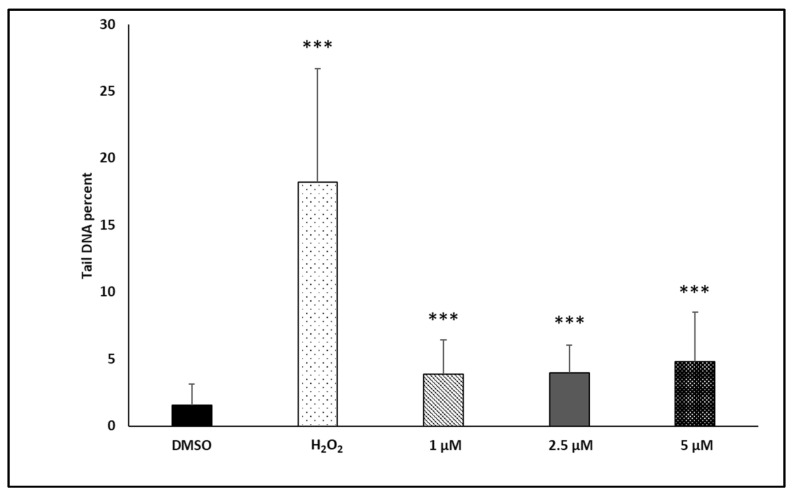
Comet assay of CCRF-CEM cells incubated with DMSO (negative control), H_2_O_2_ (positive control), or artemisinin dimer isoniazide. Comets indicative of DNA damage were observed upon treatment with H_2_O_2_ and increasing concentrations of artemisinin dimer isoniazide but not in DMSO-treated cells. Statistical significance in comparison to control (DMSO) is designated at a level of *** *p* ≤ 0.001.

The alkaline comet assay was performed to study double-strand DNA breaks, which are observable as a fluorescent, tail-like pattern of the affected cells. Treatment with H_2_O_2_ (positive control) induced an obvious pattern, indicating that DNA was damaged. Similarly, the artemisinin dimer isoniazide induced DNA damage in a dose-dependent manner, while no effect was detected after treatment with DMSO (negative control), as shown in Figure 9.

## 4. Discussion

Artemisinin-type compounds have been extensively studied for their anticancer activities. They showed promising, multitargeted effects on diverse types of cancers in vitro and in vivo. These profound activities of artemisinin and its derivatives include the induction of apoptosis, cell cycle arrest, antiangiogenic properties, the generation of reactive oxygen species, the induction of ferroptosis and necrosis, DNA double-strand breaks, and the inhibition of tumor invasion and migration [36,37,38,39]. Several cellular targets, mediators, and signaling pathways were reported to be altered by artemisinin and its derivatives. For example, several studies showed alterations in the expression of nuclear factor-kappa B (NF-κB), matrix metalloproteinases (MMPs), hypoxia-inducible factor (HIF)-1α, and vascular endothelial growth factor (VEGF) and its receptors, in addition to cell cycle and apoptosis regulators [40].

In this study, we first investigated the anticancer activity of six artemisinin dimers against CCRF-CEM cells and their corresponding multidrug-resistant CEM/ADR5000 subline. Interestingly, these artemisinin derivatives demonstrated potent growth inhibitory activities in the nanomolar range in both cell lines which were better than the reported activity of the known lead compound artemisinin or its derivative artesunate [41]. Although the artemisinin dimers possess structural similarities with the presence of two artemisinin moieties, the introduction of an extra linker moiety led to the observed variations in the IC_50_ values. All compounds were cross-resistant in CEM/ADR5000 with a variable degree of resistance from 2.1 to 49.2, indicating the relationship between a modification in the chemical structure and resistance in CEM/ADR5000. The artemisinin dimer isoniazide (ELI-XXIII-98-2) showed the best growth inhibitory activity and the lowest IC_50_ value in CCRF-CEM cells. Therefore, we selected this compound for further mechanistic analyses. 

The protooncogene c-MYC and its encoded transcription factor protein, c-MYC, are deregulated in many types of cancers. In contrast, its expression is tightly regulated in normal, healthy tissues [42]. MYC primarily functions as a transcriptional regulator, controlling genes involved in various cellular processes, such as cell division, differentiation, apoptosis, metabolism, angiogenesis, DNA repair, immunological response, protein translation, and stem cell development [43,44]. 

According to numerous in vitro and in vivo studies for cell transformation phenotypes, MYC is one of the most powerful oncogenes. It cooperates with other oncogenic factors to trigger tumorigenesis. Furthermore, MYC-induced tumors are reported to be oncogene-addicted or dependent [42]. Such an assumption is supported by data confirming that MYC suppression could reverse tumorigenesis, which was detected in a variety of tumors such as lymphoma, leukemia, epithelial tumors, and mesenchymal tumors [45,46,47,48].

The MYC transcription factor controls the expression of genes involved in cellular proliferation and growth. This transcriptional activity is mediated through DNA binding and interaction with other transcription regulators as well as cellular mediators [49]. MYC-associated factor X (MAX) is an obligate partner of c-MYC which is involved in DNA binding and MYC/MAX heterodimerization as well as functional regulation [50]. Accordingly, several small molecules with acceptable affinity to MYC/MAX have been developed. Specifically, 10058-F4 inhibited MYC/MAX heterodimerization and demonstrated cytotoxic activity and apoptosis induction activities in a variety of cancer cell lines [51,52,53].

We performed molecular docking to study the in silico binding affinities of our compound and the known c-MYC inhibitor 10058-F4, using the same grid box coordinates. Docking revealed that the artemisinin dimer isoniazide bound to c-MYC with a higher affinity than the control compound, 10058-F4. The binding of 10058-F4 was more confined to the MYC/MAX interaction site, while the artemisinin dimer isoniazide was closer to the DNA binding site. Furthermore, the superiority of the artemisinin dimer isoniazide over 10058-F4 was confirmed using a c-MYC reporter cell assay. The artemisinin dimer isoniazide significantly inhibited the activity of c-MYC in concentrations much lower than that of 10058-F4. Moreover, MST revealed an in vitro binding of the artemisinin dimer isoniazide with c-MYC with a very low K_d_ value (1.19 ± 0.24 µM), which was better than that of the control inhibitor, 10058-F4, as reported in the literature (5–13 µM) [54]. It has been reported that 10058-F4 downregulates the expression of the c-MYC protein in acute myeloid leukemia [53]. Similarly, the artemisinin dimer isoniazide significantly reduced the expression of c-MYC in microarray analyses, a qPCR, and immunoblotting. Moreover, microarray analyses as well as a qPCR revealed a clear downregulation of some genes that are involved in tumorigenesis, such as *HIFA1*, *MTOR*, and *MAPKAPK2*. Such an effect confirmed that the dimer isoniazide may act through several interacting mechanisms. 

As a proto-oncogene and transcription factor, c-MYC plays an essential role in the regulation of the cell cycle and apoptosis. c-MYC increases cellular proliferation through the upregulation of several genes required for cell cycle propagation, such as cyclins and cyclin-dependent kinases. Moreover, it suppresses the activity of cell cycle inhibitors [55]. On the other hand, c-MYC has paradoxical effects on apoptosis. While it induces apoptosis by the regulation of both pro-apoptotic and anti-apoptotic factors, c-MYC may also prevent apoptosis under certain conditions [56,57]. Such contradictory activities may explain the effects of artemisinin dimer isoniazide on c-MYC expression as well as apoptosis. We observed the induction of apoptosis; nevertheless, it was not strongly induced. Accordingly, we were interested in exploring other modes of cell death, such as autophagy.

Autophagy is an essential catabolic process in which cellular components, misfolded proteins, and damaged organelles are degraded and recycled with the aid of lysosomes. It plays a crucial role in cellular homeostasis, enabling the cells to adapt to stressful conditions. Moreover, autophagy modulates cell survival as well as cell death [58].

Upon autophagosome formation, the p62 protein functions as a receptor for the degradation of ubiquitinated substrates, interacting with LC3. Subsequently, the autophagy-related protein 4 (ATG4) cleaves LC3 to generate cellular LC3-I, which is then covalently attached to phosphatidylethanolamine on the phagophore membrane to form LC3-II. Both LC3-II and p62 are finally degraded after fusion with the lysosome. Therefore, the altered expression of both proteins could be an indicator of the activation or inhibition of autophagy [59,60,61].

Numerous studies have proven the interaction between c-MYC and autophagy regulation. In a model of non-small cell lung carcinoma (NSCLC), c-MYC switched off the onset of autophagy via the induction of miR-150, leading to increased ER stress, DNA damage, and cancer cell proliferation. Consequently, treatment with 10058-F4 reversed c-MYC- and miR-150-dependent autophagy defects and induced cytotoxicity and tumor reduction in NSCLC in vitro and in vivo [62]. Similarly, 4-O-methyl-ascochlorin concurrently downregulated c-MYC and induced autophagy in K562 leukemic cell lines [63].

In this study, we reported the induction of autophagy in CCRF-CEM cells upon treatment with the artemisinin dimer isoniazide which was seen as an upregulation of the autophagy marker LC3-II and downregulation of p62. As reported in the literature, autophagy induction could be attributed to the inhibition of both c-MYC function and expression. 

Several studies have reported the involvement of c-MYC in the regulation of DNA damage signaling. C-MYC can activate several genes involved in DNA damage response (DDR), such as BRCA2, ATM, PRKDC, and TP53. For instance, the ability to repair double-strand breaks was diminished in MYC-knockdown Hela-630 cells after ionizing radiation [64,65]. On the contrary, DNA damage led to decreased expression of c-MYC via proteasome-dependent degradation [66,67]. It has been reported that the MYC mRNA, protein level, and transcriptional activity can be inhibited by DNA-damaging drugs, such as topoisomerase II inhibitors, or by ionizing radiation in MCF-7 breast cancer cells [68,69,70]. Interestingly, we observed a similar pattern in our study. Microarray analyses showed that some DNA damage response genes controlled by c-MYC (e.g., *PRKDC*) were deregulated. The contradictory effects of c-MYC might explain the slight activation of apoptosis by the artemisinin dimer isoniazide. Moreover, the c-MYC level may be reduced directly by the artemisinin dimer isoniazide or indirectly via induction of DNA damage.

H2AX is a member of the H2A histone family, which is one of four families of histone proteins that are essential for DNA wrapping, nucleosome formation, and chromatin regulation. H2AX is characterized by the presence of a hydrophobic amino acid in its C terminal. In response to DNA double-strand breaks, H2AX is phosphorylated in Ser 139 position and hence named γ-H2AX; it is not only important as a marker for DNA damage but also as an essential player in maintaining genomic integrity [71,72]. As c-MYC participates in DNA damage response and repair mechanisms, studies have shown the correlation between c-MYC and γ-H2AX levels. After DNA damage, phosphorylated c-MYC showed a high degree of co-localization with γ-H2AX as well as phosphorylated DNA-dependent protein kinase catalytic subunit (DNA-PKcs) S2056 clusters. Accordingly, c-MYC inhibition led to double-strand breaks and, subsequently, the elevation of γ-H2AX levels [73].

Remarkably, the artemisinin dimer isoniazide significantly upregulated phosphorylated H2AX both in the microarray analyses and immunoblotting. Furthermore, this activity was confirmed by the comet assay, proving the induction of DNA damage, a mechanism that may contribute to the growth inhibitory activity of this dimer molecule.

## 5. Conclusions

**Figure 10:**
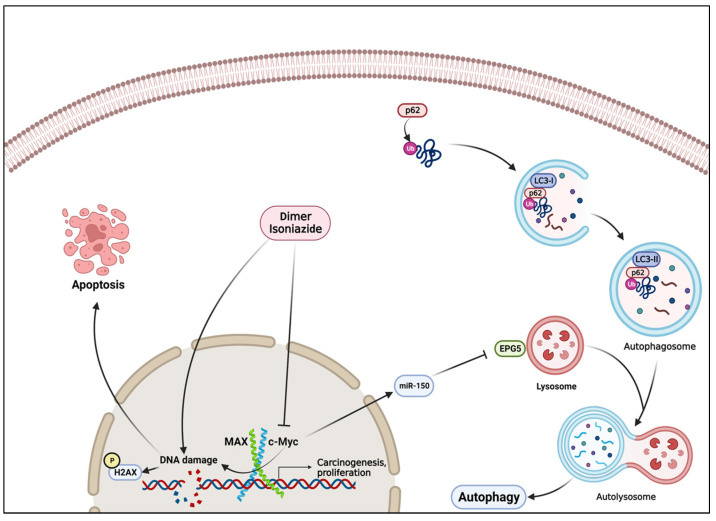
The molecular mechanism of the artemisinin dimer isoniazide. c-MYC inhibits autophagy via miR-150 and EPG axis; therefore, the inhibition of c-MYC by dimer isoniazide led to the induction of autophagy. On the other hand, dimer isoniazide may directly or indirectly—via c-MYC inhibition—cause DNA damage which, in turn, triggers apoptosis. Figure created with BioRender.com.

In conclusion, the artemisinin dimer isoniazide possesses a potent growth inhibitory activity in leukemic cell lines. This effect could be manifested by the inhibition of c-MYC activity and expression, the induction of autophagy, and the stimulation of DNA damage, as shown in Figure 10. This study emphasizes the significance of artemisinin derivatization as a potential source of effective anticancer agents and future cancer therapy. Further in vivo studies are needed to further characterize the anticancer potential of this artemisinin dimer isoniazide. 

## 6. Patents

M.A.E. and W.G. disclose the patent: US Patent US 7842,720 B2. T.E. discloses the patents: US60/619,829, ES2245248A1, and AD3503EP.

## Figures and Tables

**Table 1:** Molecular weights and IC_50_ values of wild-type, drug-sensitive CCRF-CEM and multidrug-resistant CEM/ADR5000 cells from resazurin assays of six artemisinin dimers. Shown are mean values ± standard deviation of three independent experiments, each with six parallel measurements.

Compound	Name	Molecular Weight	IC_50_ for CCRF-CEM (nM)	IC_50_ for CEM/ADR5000 (nM)	Degree of Resistance
ELI-XXIIID-98-2	Dimer isoniazide	743.4	2.5 ± 1.5	36 ± 9	14.4
ELI-XXIIID-90-2	Dimer morpholine	693.4	2.9 ± 2.0	45 ± 15	15.5
ELI-XXIIID-88-2	Dimer benzylamine	713.9	6.0 ± 0.7	295 ± 24	49.2
ELI-XXIIIB-30-4	Dimer aniline	699.8	6.0 ± 1.9	141 ± 49	23.5
ELI-XXIIIB-3-4A	Dimer oxime	637.7	14.0 ± 0.8	29.0 ± 14.0	2.1
ELI-XXIIID-13-8	Dimer piperidine	691.8	21.0 ± 10.0	233.0 ± 28.0	11.1

**Table 2:** Sequences of qPCR primers.

Gene Name	Gene Symbol	Forward Primer	Reverse Primer
c-MYC proto-oncogene	*MYC*	ACACTAACATCCCACGCTCTG	CTCGCTAAGGCTGGGGAAAG
Hypoxia-inducible factor 1 subunit α	*HIF1A*	GATCACCCTCTTCGTCGCTT	CTCAGGTGGCTTGTCAGGG
Mammalian target of rapamycin kinase	*MTOR*	TTAGAGGACAGCGGGGAAGG	TTCCTTTAATATTCGCGCGGC
Mitogen-activated protein kinase-activated protein kinase 2	*MAPKAPK2*	AAAGGTCCCTCAAACCCCAC	ATCCTCTGCTCACAACCTGG
Glyceraldehyde-3-phosphate dehydrogenase	*GAPDH*	GCTCTCTGCTCCTCCTGTTC	GACTCCGACCTTCACCTTCC

**Table 3:** Molecular docking results showing lowest binding energies, predicted inhibition constants, and amino acid interactions for artemisinin dimer isoniazide (ELI-XXIIID-98-2) and the known c-MYC inhibitor 10058-F4 (positive control).

Compound	Lowest Binding Energy (kcal/mol)	pKi (nM)	Amino Acid Interactions
Dimer isoniazide	−9.84 ± 0.3	66.46 ± 29.5	PRO938, LYS936, ARG914, LYS918, ARG919, PHE922, LYS939
10058-F4	−4.92 ± 0.01	248.39 ± 3.51	ARG925, LEU931, GLU932, GLN927, PRO929

## Data Availability

The data are contained within the article.
